# Irish Veterinary Journal reviewer acknowledgement 2013

**DOI:** 10.1186/2046-0481-67-3

**Published:** 2014-02-13

**Authors:** Michael L Doherty

**Affiliations:** 1 UCD School of Veterinary Medicine, University College Dublin, Belfield, Dublin 4, Ireland

## Abstract

**Contributing reviewers:**

The *Irish Veterinary Journal* editorial team would like to thank the following colleagues who contributed to peer review for the journal in 2013.

## 

Naveed Akhtar

Pakistan

Valeria Albanese

United States of America

Charles Bacon

United States of America

Marijke Beltman

Ireland

James Breen

United Kingdom

Ben Brilot

United Kingdom

Murray Brown

United States of America

Franz Brulisauer

United Kingdom

Bryce Buddle

New Zealand

Sean Callanan

Ireland

Joe Cassidy

Ireland

Stephanie Caston

United States of America

Peter Chapman

United States of America

Karin Darpel

United Kingdom

Theo de Waal

Ireland

Michael Diskin

Ireland

Fiona Doohan

Ireland

Edward Dubovi

United States of America

Seamus Fanning

Ireland

James Gibbons

Ireland

John Gilmore

Ireland

Eamonn Gormley

Ireland

Jeanne Gradé

Belgium

Terence Grimes

Ireland

Kei Hayashi

United States of America

Bernhard Kaltenboeck

United States of America

Niel Karrow

Canada

Lauren Lacorcia

Australia

Desmond Leadon

Ireland

Ingrid Lorenz

Ireland

Keith Macmillan

Australia

John Madigan

United States of America

Bryan Markey

Ireland

John Mee

Ireland

Patti Miller

United States of America

Stefano Nardelli

Italy

Peter Nettleton

United Kingdom

Erasmus Okine

Canada

Ronan O’Neill

Ireland

Chris Oura

Trinidad and Tobago

Felisbina Queiroga

Portugal

Sarah Randolph

United Kingdom

Charlotte Sandersen

Belgium

Horst Schirrmeier

Germany

Julie Settlage

United States of America

Robert Shiel

Australia

George Stewart

United States of America

David Suarez

United States of America

David Verner-Jeffreys

United Kingdom

Andrew Waller

United Kingdom

Keith Way

United Kingdom

Paul Whyte

Ireland

Mike Woods

Ireland

